# ATP-binding cassette subfamily B member 1 (ABCB1) and subfamily C member 10 (ABCC10) are not primary resistance factors for cabazitaxel

**DOI:** 10.1186/s40880-015-0003-0

**Published:** 2015-03-05

**Authors:** Rishil J Kathawala, Yi-Jun Wang, Suneet Shukla, Yun-Kai Zhang, Saeed Alqahtani, Amal Kaddoumi, Suresh V Ambudkar, Charles R Ashby, Zhe-Sheng Chen

**Affiliations:** Department of Pharmaceutical Sciences, College of Pharmacy and Health Sciences, St. John’s University, Queens, NY 11439 USA; Laboratory of Cell Biology, Center for Cancer Research, National Cancer Institute, National Institutes of Health, Bethesda, MD 20892 USA; Department of Basic Pharmaceutical Sciences, College of Pharmacy, The University of Louisiana, Monroe, LA 71209 USA

**Keywords:** ATP-binding cassette (ABC) transporters, ATP-binding cassette subfamily B member 1 (ABCB1), ATP-binding cassette subfamily C member 10 (ABCC10), Taxane, Paclitaxel, Cabazitaxel

## Abstract

**Introduction:**

ATP-binding cassette subfamily B member 1 (ABCB1) and subfamily C member 10 (ABCC10) proteins are efflux transporters that couple the energy derived from ATP hydrolysis to the translocation of toxic substances and chemotherapeutic drugs out of cells. Cabazitaxel is a novel taxane that differs from paclitaxel by its lower affinity for ATP-binding cassette (ABC) transporters.

**Methods:**

We determined the effects of cabazitaxel, a novel tubulin-binding taxane, and paclitaxel on paclitaxel-resistant, ABCB1-overexpressing KB-C2 and LLC-MDR1-WT cells and paclitaxel-resistant, ABCC10-overexpressing HEK293/ABCC10 cells by calculating the degree of drug resistance and measuring ATPase activity of the ABCB1 transporter.

**Results:**

Decreased resistance to cabazitaxel compared with paclitaxel was observed in KB-C2, LLC-MDR1-WT, and HEK293/ABCC10 cells. Moreover, cabazitaxel had low efficacy, whereas paclitaxel had high efficacy in stimulating the ATPase activity of ABCB1, indicating a direct interaction of both drugs with the transporter.

**Conclusion:**

ABCB1 and ABCC10 are not primary resistance factors for cabazitaxel compared with paclitaxel, suggesting that cabazitaxel may have a low affinity for these efflux transporters.

## Background

Paclitaxel is a clinically used chemotherapeutic drug, but its use can elicit resistance to various anticancer drugs in certain types of cancers [1]. The efficacy of paclitaxel can be attenuated by the overexpression of multidrug efflux transporters, altered metabolism, decreased sensitivity to apoptosis, alterations in microtubule dynamics, diminished interactions of paclitaxel with its cellular target, and genetic polymorphisms [1,2]. These aforementioned mechanisms of resistance typically produce chemotherapeutic failure. ATP-binding cassette subfamily B member 1 [ABCB1, also called multidrug resistance 1 (MDR1) or P-glycoprotein (P-gp)] and ATP-binding cassette subfamily C member 10 [ABCC10, also called multidrug resistance protein 7 (MRP7)] have been well characterized in terms of their capacities to confer resistance to paclitaxel [1,3-5]. The human ABCB1 transporter, a product encoded by the *ABCB1* gene, which is localized to chromosome 7p21, is the first identified mammalian ATP-binding cassette (ABC) transporter [6,7]. The ABCB1 transporter has a molecular weight of 170 kDa and comprises two transmembrane-binding domains (TMD1 and TMD2) and two nucleotide-binding domains (NBD1 and NBD2) [4,8]. The human ABCC10 transporter is encoded by the *ABCC10* gene, which is localized to chromosome 6p21.1 [9,10]. The ABCC10 transporter is a 171-kDa protein, containing three membrane-spanning domains (MSD1, MSD2, and MSD3) and two NBDs. It belongs to the class of long ABCCs that includes ABCC1, ABCC2, ABCC3, and ABCC6 [11].

Cabazitaxel is a new semisynthetic taxane approved for use by the United States Food and Drug Administration and is derived from 10-deacetyl-baccatin III, which is extracted from European yew needles [12]. Mechanistically, cabazitaxel exerts its cytotoxic effects by 1) binding to tubulin and promoting its assembly into microtubules while simultaneously inhibiting microtubule disassembly and 2) stabilizing microtubules, resulting in the inhibition of mitotic and interphase cellular functions [13]. It has been postulated that cancer cells expressing ABCB1 become resistant to taxanes [14]. Apart from ABCB1, the *ABCC10* transcript has also been detected in several adenocarcinomas, including breast, ovarian, and lung tumors. This is of potential interest because the latter tumors are treated with taxanes. The *ABCC10* transcript and ABCC10 protein were reported to be induced by vincristine exposure in two salivary gland adenocarcinoma cell lines that are cross-resistant to docetaxel, and the *ABCC10* transcript was reported to be increased in MCF7 cells by exposure to doxorubicin. Moreover, ABCC10 is induced by paclitaxel in a non-small cell lung cancer cell line [3,15]. Our previous studies reported that ABCB1 and ABCC10 confer resistance to two classes of drugs that target microtubules: Vinca alkaloids and taxanes [1,16,17]. It was therefore of interest to determine if ABCB1 and ABCC10 might also confer resistance to new anti-microtubule drugs. Thus, we hypothesized that ABCB1 and ABCC10 may confer differential resistance to paclitaxel and cabazitaxel. In this study, we determined the sensitivity of ABCB1- and ABCC10-overexpressing cells to paclitaxel and cabazitaxel *in vitro*.

## Methods

### Materials

Cabazitaxel was purchased from MedChemexpress (Manmouth Junction, NJ, USA). Paclitaxel was purchased from Tocris Bioscience (Ellisville, MO, USA). Dulbecco’s modified Eagle’s medium (DMEM), fetal bovine serum (FBS), phosphate-buffered saline (PBS), 10,000 IU/mL penicillin and 10,000 μg/mL streptomycin, and 0.25% trypsin were purchased from HyClone (Waltham, MA, USA). 3-(4,5-Dimethylthiazol-yl)-2,5-diphenyltetrazolium bromide (MTT), dimethyl sulfoxide (DMSO), ammonium molybdate, 2-(N-morpholino) ethanesulfonic acid (MES) hydrate, antimony potassium tartrate, sodium azide, and N-methyl-D-glucamine were obtained from Sigma Chemical Co. (St. Louis, MO, USA). Potassium phosphate, ethylene glycol tetraacetic acid (EGTA), and adenosine triphosphate (ATP) were products of AMRESCO (Solon, OH, USA). Sulfuric acid solution (37 N) was purchased from Fisher Scientific (Pittsburgh, PA, USA). KCl was purchased from Avantor Performance Materials (Center Valley, PA, USA). Ouabain was purchased from Enzo Life Sciences, Inc. (Farmingdale, NY, USA). Dithiothreitol was purchased from Promega Corporation (Madison, WI, USA). MgCl_2_ was purchased from EMD Millipore (Billerica, MA, USA). Ascorbic acid was purchased from VWR International (West Chester, PA, USA). Sodium orthovanadate was purchased from Alfa Aesar (Ward Hill, MA, USA). The OPSYS microplate reader was purchased from Dynex Technologies (Chantilly, VA, USA).

### Cell lines

The ABCB1-overexpressing KB-C2 cell line was established by a step-wise exposure of KB-3-1 cells, a parental human epidermoid carcinoma cell line, to increasing concentrations (up to 2 μg/mL) of colchicine [18]. The LLC-PK1 porcine renal epithelial cells were transfected with wild-type human *ABCB1* cDNA plasmid as previously described, and the stable transfectant cell line was named LLC-MDR1-WT [19]. HEK293/pcDNA3.1 and HEK293/ABCC10 cells were generated by transfecting HEK293 cells with an empty vector and an ABCC10 expression vector, respectively [20]. We thank Dr. Shinichi Akiyama (Kagoshima University, Japan) for the KB-3-1 and KB-C2 cell lines, Dr. Michael M. Gottesman (NCI, NIH, USA) for the LLC-PK1 and LLC-MDR1-WT cell lines, and Dr. Gary D. Kruh (University of Illinois at Chicago, IL, USA) for the *ABCC10* plasmid.

### Drug sensitivity

To determine the drug sensitivities of the previously described ABCB1-overexpressing KB-C2 cells, LLC-MDR1-WT cells, and ABCC10-overexpressing HEK293/ABCC10 cells, with KB-3-1, LLC-PK1, and HEK293/pcDNA3.1 cells as the respective controls [1,16], a modified MTT assay was performed [1,21]. Approximately 4,000 KB-3-1 cells, 7,000 KB-C2 cells, and 5,000 LLC-PK1, LLC-MDR1-WT, HEK293/pcDNA3.1, and HEK293/ABCC10 cells were seeded in 180 μL of medium in each well of 96-well plates. After incubating for 24 h at 37°C, 20 μL of paclitaxel or cabazitaxel (0.01 to 10 μmol/L) was added. Subsequently, cells treated with paclitaxel or cabazitaxel in DMEM supplemented with 10% FBS were incubated at 37°C for 72 h. After 72 h, 20 μL MTT (4 mg/mL) was added to each well. The cells were incubated at 37°C for another 4 h. The MTT with medium was removed, and 100 μL of DMSO was added to each well. The absorbance was measured at 570 nm by an Opsys microplate reader (Dynex Technologies, VA, USA). The degree of resistance was calculated by dividing the 50% inhibition concentration (IC_50_) as calculated using the Bliss method for drug-resistant cells (KB-C2, LLC-MDR1-WT, and HEK293/ABCC10) by that of the parental drug-sensitive cells (KB-3-1, LLC-PK1, and HEK293/pcDNA3.1), respectively. Each MTT assay was run in triplicate.

### ABCB1 ATPase assay

The ABCB1 transporter uses energy derived from the hydrolysis of ATP to efflux their substrates across the membrane against a concentration gradient; thus, the ATP consumption reflects the ATPase activity of the transporter. The vanadate (Vi)-sensitive ATPase activity of ABCB1 in the membrane vesicles of High Five insect cells [(His)-6-tagged ABCB1 expressed in *Trichoplusia ni* cells using the recombinant baculovirus system and purified by metal affinity chromatography] was measured as previously described [22,23]. The membrane vesicles (10–20 μg protein/reaction) were incubated in ATPase assay buffer (50 mmol/L MES-Tris, pH 6.8, 50 mmol/L KCl, 5 mmol/L sodium azide, 1 mmol/L EGTA, 1 mmol/L ouabain, 2 mmol/L dithiothreitol, and 10 mmol/L MgCl_2_) at 37°C for 5 min with or without 0.3 mmol/L vanadate. The membrane vesicles in ATPase assay buffer were incubated with different concentrations (0–10 μmol/L) of paclitaxel or cabazitaxel at 37°C for 3 min, and then 5 mmol/L ATP was added at 37°C. After 20 min of incubation, the reaction was terminated by adding 0.1 mL of 5% SDS solution. The amount of Pi released was quantified at 800 nm using a Bio-Rad SmartSpec Plus Spectrophotometer (Hercules, CA, USA) as previously described [23,24].

### Statistical analyses

All experiments were repeated at least three times. The differences between runs were assessed using the two-tailed Student’s *t*-test, and statistical significance was determined at *P* < 0.05. Microsoft Office Excel 2010, licensed from Microsoft (Redmond, WA, USA), was used for data processing and analysis.

## Results

### Cytotoxicity of paclitaxel and cabazitaxel in ABCB1- and ABCC10-overexpressing cells

**Figure 1:**
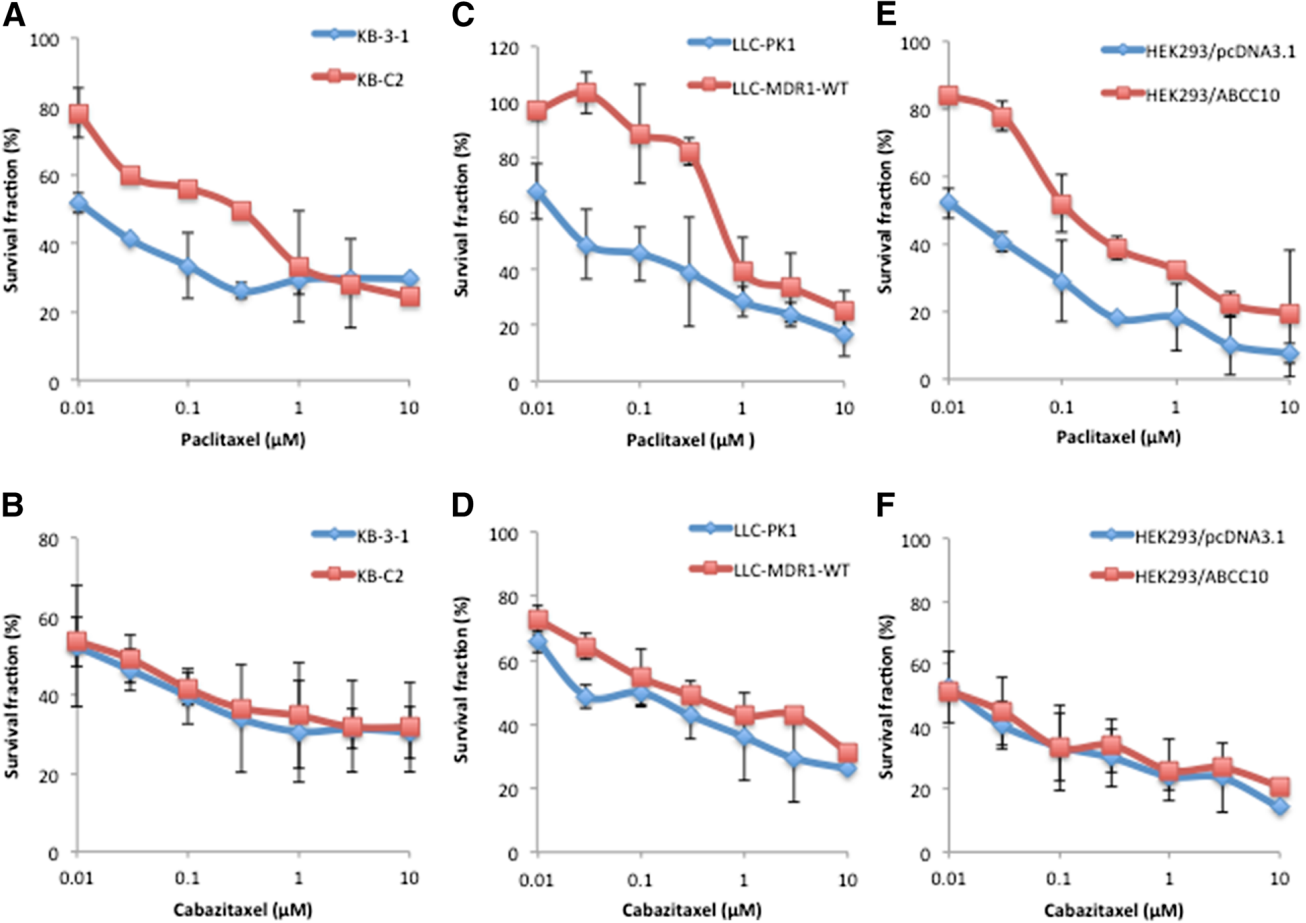
**Cytotoxicity of paclitaxel and cabazitaxel in ABCB1- and ABCC10-overexpressing cells.** ABCB1, ATP-binding cassette subfamily B member 1; ABCC10, ATP-binding cassette subfamily C member 10. The cytotoxicity of paclitaxel and cabazitaxel was determined by the MTT assay in ABCB1-overexpressing KB-C2 and LLC-MDR1-WT cells, ABCC10-overexpressing HEK293/ABCC10 cells, and their parental KB-3-1, LLC-PK1, and HEK293/pcDNA3.1 cells. Error bars indicate the standard deviation (SD). Significantly elevated resistance of KB-C2 **(A)** and LLC-MDR1-WT cells **(C)** to paclitaxel, none or low-level resistance of KB-C2 **(B)** and LLC-MDR1-WT cells **(D)** to cabazitaxel, significantly elevated resistance of HEK293/ABCC10 cells to paclitaxel **(E)**, and no resistance of HEK293/ABCC10 cells to cabazitaxel **(F)** are observed as compared with those of their parental cells.

As shown in Table 1, significantly elevated resistance to paclitaxel was observed for the ABCB1-overexpressing, drug-selective KB-C2 cells and *ABCB1*-transfected LLC-MDR1-WT cells, which exhibited 21.2- and 25.6-fold resistance as compared with those of the parental KB-3-1 and LLC-PK1 cells, respectively; weakly elevated resistance to cabazitaxel was observed for KB-C2 and LLC-MDR1-WT cells, which exhibited 2.2- and 9.1-fold resistance as compared with those of KB-3-1 and LLC-PK1 cells, respectively. In addition, the resistance of ABCC10-overexpressing cells to paclitaxel and cabazitaxel was analyzed. The *ABCC10*-transfected HEK293/ABCC10 cells exhibited a 9.3-fold resistance to paclitaxel and 1.0-fold resistance to cabazitaxel as compared with those of HEK293/pcDNA3.1 cells. Representative concentration-response curves for paclitaxel and cabazitaxel are shown in Figure 1.

**Table 1:** **Drug sensitivities of ABCB1- and ABCC10-overexpressing cells**

**Cell line**	**IC**_**50**_ **of paclitaxel (nmol/L)**	**FR**	***P*** **value**	**IC**_**50**_ **of cabazitaxel (nmol/L)**	**FR**	***P*** **value**
**KB-3-1**	11.7 ± 2.7	21.2	0.031	13.9 ± 5.5	2.2	**0.114**
**KB-C2**	249.4 ± 60.9			31.2 ± 7.1		
**LLC-PK1**	30.5 ± 2.2	25.6	0.004	27.9 ± 0.7	9.1	**0.001**
**LLC-MDR1-WT**	781.9 ± 70.2			255.1 ± 12.2		
**HEK293/pcDNA3.1**	12.8 ± 1.4	9.3	0.010	12.5 ± 2.1	1.0	**0.799**
**HEK293/ABCC10**	119.2 ± 15.7			13.1 ± 1.8		

IC_50_, 50% inhibition concentration; FR, fold resistance. All IC_50_ values are expressed as the mean ± standard deviation (SD). FR was calculated by dividing the IC_50_ values of paclitaxel or cabazitaxel for resistant cells (KB-C2, LLC-MDR1-WT, or HEK293/ABCC10 cells) by those for the parental sensitive cells (KB-3-1, LLC-PK1, or HEK293/pcDNA3.1 cells, respectively). The values in the table are representative of at least 3 independent experiments.

### Effects of paclitaxel and cabazitaxel on ABCB1 ATP hydrolysis

**Figure 2:**
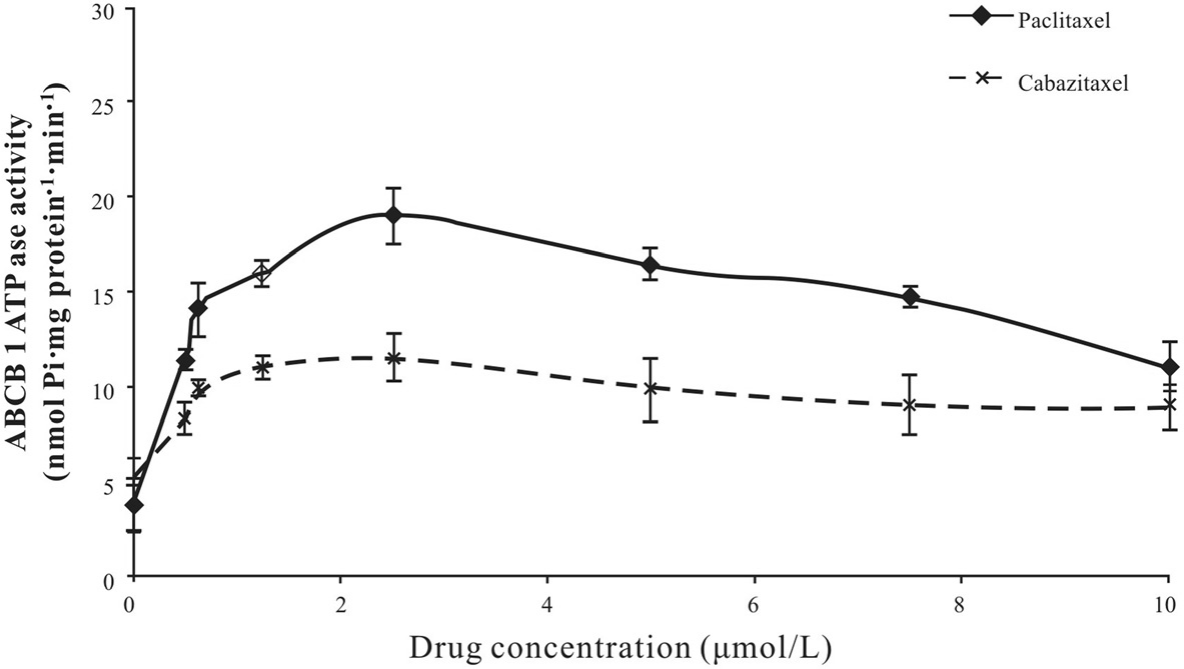
**Stimulation of ABCB1 ATPase activity by paclitaxel and cabazitaxel.** Vanadate-sensitive ABCB1 ATPase activity was stimulated in the presence of the indicated concentrations of paclitaxel and cabazitaxel. Error bars indicate the SD.

Paclitaxel and cabazitaxel stimulated the ATPase activity of ABCB1 (Figure 2), suggesting that paclitaxel and cabazitaxel interact at the drug substrate-binding site and increase the ATPase activity of ABCB1. However, less stimulation of the ATPase activity of ABCB1 was observed with cabazitaxel as compared with paclitaxel, suggesting that cabazitaxel has a lower affinity for ABCB1, consistent with the cytotoxicity results.

## Discussion

In the present study, ABCB1-overexpressing, drug-selective KB-C2 cells and *ABCB1*-transfected LLC-MDR1-WT cells showed low resistance to cabazitaxel. ABCC10-overexpressing HEK293/ABCC10 cells exhibited no resistance to cabazitaxel as compared with their resistance to paclitaxel. Furthermore, cabazitaxel stimulated the ATPase activity of ABCB1 to a lesser magnitude than paclitaxel.

The present analysis of ABCB1 and ABCC10 efflux transporters provides important and new information on the resistance profile related to these pumps. A notable feature of ABCB1 and ABCC10 that emerged from this line of investigation is that these pumps are able to confer little or no resistance to cabazitaxel. Of the microtubule-stabilizing drugs recently used in clinical development, cabazitaxel is the most advanced one [12]. As such, cabazitaxel exhibits a low affinity for ABCB1 or any previously tested drug efflux pump [25]. Our results suggest that the differential resistance properties of ABCB1- and ABCC10-overexpressing cells to paclitaxel and cabazitaxel may be attributed to structural differences of these drugs, although this remains to be determined.

Duran *et al.* [26] reported several potential biomarkers for predicting cabazitaxel resistance in cell lines that did not express the ABCB1 protein; these biomarkers included reduced breast cancer type 1 susceptibility protein (BRCA1) expression and increased class III β-tubulin isotype expression. Moreover, alterations in microtubule dynamics and induction of the epithelial-mesenchymal transition have been linked to the resistance mechanism of cabazitaxel [26]. Thus, it is possible that the drug-resistant cell lines used in our study may express the aforementioned markers and confer resistance to cabazitaxel. Therefore, future experiments will be directed toward determining the mechanisms that mediate cabazitaxel resistance by various methods, such as measuring the cellular retention of cabazitaxel in cell lines that are positive and negative for ABC transporters.

## Conclusions

In conclusion, cabazitaxel, unlike paclitaxel, sensitizes ABCB1- and ABCC10-overexpressing cells. It is possible that cabazitaxel is a low-affinity substrate of ABCB1 and ABCC10 and that these transporters may not play a role in the development of drug resistance in the clinic. However, further preclinical studies are warranted to establish this fact.
